# START NOW - a comprehensive skills training programme for female adolescents with oppositional defiant and conduct disorders: study protocol for a cluster-randomised controlled trial

**DOI:** 10.1186/s13063-016-1705-6

**Published:** 2016-12-01

**Authors:** Linda Kersten, Martin Prätzlich, Sandra Mannstadt, Katharina Ackermann, Gregor Kohls, Helena Oldenhof, Daniel Saure, Katrin Krieger, Beate Herpertz-Dahlmann, Arne Popma, Christine M. Freitag, Robert L. Trestman, Christina Stadler

**Affiliations:** 1 Department of Child and Adolescent Psychiatry/Department of Psychology, University Psychiatry Clinics Basel/University of Basel, Basel, Switzerland; 2 Department of Child and Adolescent Psychiatry, Psychosomatics and Psychotherapy, University Hospital Frankfurt, Goethe-Universität, Frankfurt am Main, Germany; 3 Department of Child and Adolescent Psychiatry, Psychosomatics and Psychotherapy, RWTH Aachen University, Aachen, Germany; 4 Department of Child and Adolescent Psychiatry, de Bascule, University of Amsterdam/VUmc, Amsterdam, The Netherlands; 5 Institute of Medical Biometry and Informatics, Department Medical Biometry, University of Heidelberg, Heidelberg, Germany; 6 Coordination Centre for Clinical Trials, University Hospital Heidelberg, Heidelberg, Germany; 7 Correctional Managed Health Care, UConn Health, Farmington, CT USA

**Keywords:** Randomised controlled trial, Treatment, Adolescents, Females, Residential care, Conduct disorder, Oppositional defiant disorder, Skills training, Emotion regulation

## Abstract

**Background:**

In Europe, the number of females exhibiting oppositional defiant disorder (ODD) and conduct disorder (CD) is growing. Many of these females live in youth welfare institutions. Consequently, there is a great need for evidence-based interventions within youth welfare settings. A recently developed approach targeting the specific needs of girls with ODD and CD in residential care is START NOW. The aim of this group-based behavioural skills training programme is to specifically enhance emotional regulation capacities to enable females with CD or ODD to appropriately deal with daily-life demands. It is intended to enhance psychosocial adjustment and well-being as well as reduce oppositional and aggressive behaviour. We present the study protocol (version 4.1; 10 February 2016) of the FemNAT-CD intervention trial titled ‘Group-Based Treatment of Adolescent Female Conduct Disorders: The Central Role of Emotion Regulation’.

**Methods/design:**

The study is a prospective, confirmatory, cluster-randomised, parallel-group, multi-centre, randomised controlled trial with 128 institutionalised female adolescents who fulfil the diagnostic criteria of ODD and/or CD. Institutions/wards will be randomised either to provide the 12-week skills training as an add-on intervention or to provide treatment as usual. Once the first cycle is completed, each institution will run a second cycle with the opposite condition. Primary endpoints are the pre-post change in number of CD/ODD symptoms as assessed by a standardised, semi-structured psychiatric interview (Kiddie Schedule for Affective Disorders and Schizophrenia for School-Age Children–Present and Lifetime, CD/ODD section) between baseline and the end of intervention, as well as between baseline and a 3-month follow-up point. Secondary objectives include pre-post change in CD/ODD-related outcome measures, most notably emotional regulation on a behavioural and neurobiological level after completion of START NOW compared with treatment as usual.

**Discussion:**

To our knowledge, this study is the first to date to systematically investigate the effectiveness of an adapted integrative psychosocial intervention designed for female adolescents with ODD and CD in youth welfare settings.

**Trial registration:**

German Clinical Trials Register (DRKS) identifier: DRKS00007524. Registered on 18 December 2015 and with the World Health Organisation International Clinical Trials Registry Platform.

**Electronic supplementary material:**

The online version of this article (doi:10.1186/s13063-016-1705-6) contains supplementary material, which is available to authorized users.

## Background

Across European countries, an increase in mental health problems, along with a growing rate of violence and aggression in youths, can be observed [1]. Children and adolescents with conduct problems, including aggression, represent a highly heterogeneous group. From a diagnostic perspective, aggressive and oppositional behaviour has been subsumed under the diagnoses conduct disorder (CD) and oppositional defiant disorder (ODD) [2]. The heterogeneity is reflected not only by a great variety of symptoms ranging from impulsive, hot-tempered quarrels or stealing to acts of cruelty to animals or people but also by the extent of co-morbidity (e.g., attention-deficit disorder, depression, anxiety disorders, learning difficulties, posttraumatic stress disorder). Furthermore, research indicates that especially children and adolescents with ODD and CD bear a high risk for maladjustment and the persistence of aggressive and antisocial behaviour symptoms into adulthood [3–6]. In that sense, youth with ODD or CD represent a highly vulnerable group with a significant risk and cost, not only to themselves but also to their immediate surroundings and ultimately to society as a whole.

Although especially CD has long been considered a male-typical disorder (with male-to-female prevalence ratio estimates ranging from 2:1 to 8:1 [7]), recent research suggests rising and rather high prevalence rates of about 1–3% among girls and female adolescents [8–10]. Conduct problems (including ODD or sub-clinical symptoms) are observed in approximately 14% of girls and 16% of boys in Europe [11]. Not surprisingly, prevalence rates of specific populations, such as incarcerated females, are considerably higher, ranging between 32% and 73% [12]. Although the number of females exhibiting ODD and CD is growing, the majority of studies on therapeutic treatment options for CD have been focused primarily on male subjects, despite strong evidence that the course and overall psychosocial adjustment problems associated with CD are often more severe in females than in males [7, 13]. Sequelae such as school failure, substance abuse, delinquency, child prostitution, and early pregnancy (often associated with poor parenting skills) pose serious problems on an individual as well as a societal level.

The development of ODD and CD is generally seen to be caused by an interplay between adverse psychosocial and neurobiological factors that can ultimately result in severely impaired emotional regulation capacities [14]. Patients with ODD and patients with CD are characterised predominantly by impulsive-aggressive behaviour that is often associated with high emotional vulnerability exemplified by hypervigilant behaviour in response to neutral stimuli. In addition, patterns of thought that include automatic appraisals and distortions of attribution are often found. On top of that, adolescents with conduct problems frequently experience interpersonal difficulties, which are oftentimes associated with high levels of psychological distress. For these youth, a therapeutic approach focused on stress tolerance, mindfulness, emotional regulation, interpersonal skills and adequate handling of emotions is well-suited and directly tailored to their needs [15]. As for a specific sub-group of adolescents with CD that can be best described as callous-unemotional with associated features, such as a lack of emotional responsivity, lack of empathy, extreme patterns of thought (e.g., black-and-white thinking), and interpersonal difficulties without any associated psychological distress, different kinds of therapeutic strategies seem to be needed. Here, interventions focused on enhancing empathic capacities, moral reasoning and perspective-taking are required [15]. So far, research indicates that, next to individual skills training, adolescents with ODD/CD benefit immensely from therapeutic approaches that involve their direct environment [16, 17]. More specifically, children with disruptive behaviours primarily benefit from parent-focused interventions, whereas adolescents with externalising behaviours respond very well to multi-modal approaches [16].

### The intervention

One specific approach that has accumulated support for use with forensic patient populations and appears very suitable for the treatment of female adolescents with ODD/CD is START NOW [18]. Developed at the Department of Correctional Managed Health Care, University of Connecticut, USA, the skills programme was evaluated at multiple correctional facilities throughout the State of Connecticut [18]. In its original format, START NOW is a manualised, group-based skills training programme designed for use within correctional institutions. The focus of the intervention is on (1) increasing self-control over impulses, (2) enhancing emotion recognition and regulation, (3) making judgements and decisions on the basis of consequences and (4) improving stress management and coping skills [19]. START NOW is an integrative evidence-informed intervention based on several therapeutic approaches. These therapeutic elements were chosen because of research findings as well as suitability for the target population (i.e., patients with behaviour disorders) and for integration with other aspects of the planned treatment. More specifically, the programme combines aspects of cognitive behavioural therapy (CBT), motivational interviewing (MI), dialectical behaviour therapy (DBT) and trauma-sensitive care. The subsequent sections outline the different elements that comprise START NOW in more detail and elaborate on their relevance with regard to the target population.

### Cognitive behavioural therapy

CBT represents the theoretical underpinning of the intervention. The programme integrates functional analysis to help group members break down behaviour patterns and understand their own behaviour in the context of its antecedents and consequences. In addition, CBT serves to help patients understand how their own conceptualisation of events is connected to their mood and behaviour. Understanding these cognitive processes increases patients’ control and understanding of their behaviour. Furthermore, it facilitates recognition and coping with potential triggers, the modification of cognitions and attitudes, as well as the replacement of problematic behaviours with alternate conduct. Within the CBT framework, new skills are being taught and practiced within as well as between sessions (including role-playing, problem-solving, group discussions or practice exercises) to facilitate transfer [20].

A meta-analysis of outcomes from 26 research articles addressing the effectiveness of group therapy with adult offenders [21] revealed positive effects of CBT, including improved interpersonal functioning, improved self-esteem and anger management, as well as a decrease in feelings of anxiety and in disciplinary sanctions. There is also long-standing support for using CBT-based approaches with adolescents who exhibit externalising problem behaviours [22–25]. Furthermore, evidence suggests that a therapeutic group approach incorporating practical issues in daily-life situations is well-accepted in adolescents and may show high effectiveness even in severely aggressive youth [26]. In addition, CBT has been shown to be feasible for use with counsellors: A training programme using treatment manuals resulted in highly skilled implementations, high levels of counsellors’ self-reported confidence to use the intervention effectively, and high satisfaction ratings [27].

### Motivational interviewing

MI forms an additional part of the START NOW programme. It is seen as a useful complement to CBT because it represents a client-centred approach that directly addresses a patient’s ambivalence and facilitates motivation for change [28]. In this sense, MI helps to provide the basis for the patient’s willingness to engage in the intervention [29, 30], whereas CBT provides the specific tools and strategies to change problematic behaviours. Adolescents with ODD or CD do not tend to show much insight into the degree of inadequacy of their misconduct and as a result do not present with a great motivation for change. Therefore, a therapeutic stance that is marked with validation and empathy on the one hand, and the exploration of dysfunctional behaviours and their associated negative consequences on the other hand, is a feasible treatment strategy also for the treatment of adolescents with disruptive behaviour disorders [31]. START NOW uses several MI aspects, such as the view that ambivalence toward change is common and normal. Furthermore, discussions and exercises stimulating change talk and designed to overcome potential ambivalence are provided. Finally, self-efficacy and positive behaviours are evoked and reinforced throughout the whole intervention [32].

In the past, there have been other effective interventions that have combined the two approaches [33, 34]. The literature reveals positive indications for the use of MI with adolescents: Effects have been found in the areas of substance use [35, 36], weight-related problems [37, 38] and diabetes [39]. In a cluster-randomised trial investigating the effectiveness of single-session MI intended to reduce adolescent substance use behaviour, for instance, researchers found moderate to large effect sizes [36]. Furthermore, MI has been linked to better treatment retention rates mostly in adult populations in various settings [40–42].

### Cognitive modifications

Because the original format was designed for use with correctional populations, the developers decided to include cognitive modifications of the regular clinical approach in response to studies indicating that high numbers of incarcerated individuals have sustained traumatic brain injuries (TBIs) [43], as well as studies revealing low effectiveness of interventions that do not take such impairments into account [44]. Regarding the presence of TBI in adolescents exhibiting disruptive behaviour disorders, research has shown high prevalence rates [45]. In a study of the incidence of TBI among 720 delinquent adolescents in youth services institutions throughout the State of Missouri, USA, investigators found incidence rates of self-reported lifetime TBI as high as 18.3%, meaning nearly one in five adolescents reported having sustained a TBI at some point in their lives [45]. Because TBI has been linked to reduced verbal capacities, school problems and concentration problems [46], START NOW was designed to address such limitations by using participant workbooks written at a fifth-grade reading level, with little jargon, using many pictures and repetitions of key concepts and integrating a lot of practice exercises and group discussions to enhance understanding and recollection.

### Dialectical behaviour therapy

START NOW further includes some principles that are compatible with DBT. The therapy, which was originally developed for patients with borderline personality disorder [47], has been increasingly applied to forensic sample populations living in residential care settings or correctional facilities [48, 49]. Fundamental to the development of DBT is a biosocial theory that conceptualises a patient’s emotional dysregulation as the result of a biological tendency toward emotional vulnerability in conjunction with an invalidating rearing environment [50]. Within START NOW, two key concepts of DBT are found: (1) emphasis on dialectics and (2) mindfulness. The balance between validation and confrontation mainly represents the so-called dialectical relationship work that can also affect the degree of motivation for change as well as the willingness to explore new behaviour patterns [51]. Mindfulness stemming from Eastern spiritual practices was first and innovatively combined with CBT strategies within DBT [52]. Within START NOW, a subset of mindfulness exercises is offered, ranging from interactive exercises to inner mindfulness practices that involve paying close attention to one’s own body. Adolescents are encouraged throughout the programme to try each type of exercise and decide for themselves what kind of mindfulness practice best works for them.

Applying DBT strategies to settings where there is a high prevalence of ODD and CD is highly reasonable because this target population shows many of the same risk factors for the development of emotion dysregulation as do borderline patients [49, 53]. The first evidence that DBT is likely to be effective in females with CD symptoms has already been shown [54–56]. Nelson-Gray and colleagues [54], for instance, found significant reductions in externalising and internalising symptoms in an outpatient sample of youth with ODD. With regard to mindfulness specifically, a randomised controlled trial with 228 non-clinical first- to third-grade students showed significant improvements in terms of text anxiety, social skills and selective attention [57]. The use of mindfulness techniques with adolescents with externalising problems has also been investigated in a handful of studies, and to date the results are encouraging, with small to medium effect sizes, with regard to aggressive, non-compliant acts; social behaviour; attention; and subjective happiness [58–60].

### Trauma-sensitive care

START NOW integrates aspects of trauma-sensitive care. This approach includes the central idea that dysfunctional behaviour can emerge as a result of an individual’s adjustment to adverse, highly stressful life circumstances. In this sense, many kinds of currently problematic behaviours may be understood as initially adaptive coping strategies in response to negative situations. In the long term, however, those adaptive strategies may become associated with a preponderance of negative consequences. START NOW specifically uses some concepts of Trauma Affect Regulation: Guide for Education and Therapy [61], including focusing exercises as well as self-monitoring. Research indicates that adolescents in residential care settings represent a population with a high incidence of trauma histories and trauma symptoms. In one study of different types of maltreatment experiences over and above the incidence of conduct problems in female adolescents in out-of-home care, researchers identified a large proportion of the sample reporting severe to extreme maltreatment experiences, ranging from 20% for sexual abuse to 33% for emotional abuse [62]. Furthermore, these maltreatment experiences were shown to be connected to trauma-related symptoms in emerging adulthood. These findings speak to the relevance of using trauma-sensitive interventions for maltreated adolescents to help them connect their current symptoms with traumatic past experiences.

### The context

From the perspective of the social worker, adolescents’ behavioural dysregulation and persistent tendency to test boundaries, along with their lack of insight into their problematic situation, trigger anger, disappointment and helplessness [53]. It is therefore necessary to introduce a multi-modal concept that integrates all involved professions and parties to enhance communication, transparency and support and to decrease stress levels that originate from the challenges of dealing with adolescents with ODD/CD [53]. Because of the decreased chance of generalisation of the taught skills, in vivo coaching seems most adequate to help ensure that youth with ODD and CD will practice newly introduced strategies consistently in daily-life situations [15]. Unfortunately, to date, there is no study in which researchers have specifically investigated the effectiveness of a skills training programme combining the above-listed therapeutic elements for the treatment of institutionalised female adolescents with ODD or CD.

### Effectiveness of START NOW

A treatment evaluation study of the original START NOW intervention in the United States showed that this approach is effective for difficult-to-manage, impulsive and aggressive detainees [63]. Analyses of a mixed-gender sample of inmates (*n* = 946 participation events) indicated that START NOW was effective in reducing the number of disciplinary reports received. More specifically, a dose-response relationship was found: Zero-inflated negative binomial regression analyses indicated that each additional session was associated with a 5% reduction in the incidence rate of disciplinary infractions. Furthermore, participation in the skills training yielded high satisfaction rates and reduced psychiatric hospitalisation days [19, 64]. It was therefore concluded that the intervention programme represents a viable treatment option for reducing problem behaviour in incarcerated populations.

### The present study

Within the framework of the FP7 project Neurobiology and Treatment of Adolescent Female Conduct Disorder: The Central Role of Emotion Processing (FemNAT-CD; see http://www.femnat-cd.eu/ for more information), a European multi-disciplinary study, the aim of the present study is to evaluate the effects of an adapted, gender-specific START NOW intervention for females with ODD/CD living in youth welfare institutions. To our knowledge, no prior study has evaluated the effects of such a CBT-based intervention combining aspects of multiple other therapeutic approaches for this specific population in the youth welfare sector. The adaptation of the original START NOW intervention still employs the same underlying principal components of the skills training, but it uses a slightly modified framework (i.e., individual sessions on top of group sessions to enhance personal understanding and relevance of content; shortened length of the programme due to frequent temporary, time-constrained placements) and adjusted content (i.e., illustrations and theme stories tailored to teenage girls’ interests). Furthermore, the intervention will be conducted by staff members of the respective youth welfare institution. Intensive pre-training and supervision, including fidelity monitoring, will be provided. The intervention comprises weekly group sessions (14 sessions, 90 minutes each) and individual sessions (45 minutes each). The aim of the individual sessions is to ensure the transfer of the content of the group training into the personal, daily routines of the participants. Girls are encouraged to practice the skills introduced during the START NOW training between sessions in their daily social interactions with the aid of the responsible social worker. In addition, parents will be sent information on START NOW and information regarding efficient parenting strategies and communication skills with adolescents. In essence, key aspects of the START NOW skills training have remained the same within the adapted version and are well-suited for the target population that can, similarly to the target population of the original format, be described by emotional regulation deficits, many invalidating and traumatic relationship experiences, high impulsivity and a low motivation for change.

### Hypotheses

On the basis of the literature, it is hypothesised that female adolescents participating in the add-on START NOW programme will show a greater reduction of aggressive symptoms and co-morbid psychopathology as well as enhanced emotional regulation as compared with adolescents undergoing treatment as usual (TAU). The primary outcome measure refers to the change in number of ODD/CD symptoms endorsed over time as assessed using a semi-structured psychiatric interview (Kiddie Schedule for Affective Disorders and Schizophrenia for School-Age Children–Present and Lifetime [K-SADS-PL], sections ODD/CD) with the female adolescent and her respective social worker.

Regarding secondary outcomes, it is hypothesised that START NOW will result in a reduction of co-morbid psychopathology and an enhancement of life satisfaction and emotional regulation skills compared with TAU alone. The predictive value of psychosocial and neurobiological as well as specific personality variables will be investigated. The question of who benefits most from intervention programmes (and who does not) is of major interest to offer effective and tailored intervention strategies. There is accumulating evidence that patients with CD with elevated callous-unemotional traits profit less from current interventions [14, 65]. This might be due to a biologically determined hyporesponsiveness to distress cues that might interfere with social learning [66]. We thus hypothesise that reduced emotional responsiveness/elevated callousness is predictive of low intervention success in females with CD/ODD. Accordingly, we will focus on change in heart rate variability (HRV) because HRV has been identified as an important correlate of emotional regulation difficulties and treatment response [67]. In addition, in a sub-sample, we will evaluate change in neural functional correlates using a functional paradigm specifically assessing emotional regulation capacities under emotionally distressing conditions [68–71]. Further, we expect that the intervention comprising staff training and regular supervision will reduce self-reported stress and symptoms of burn-out in involved social workers.

## Methods/design

The present study is a prospective, confirmatory, cluster-randomised, multi-centre, international phase III trial with two parallel groups (intervention vs. TAU) and three trial phases (baseline assessment, 12 weeks of treatment, 12 [±1] weeks of follow-up observation). The intervention condition is incorporated into the usual standard care within residential care (START NOW + TAU) vs. TAU. A randomised controlled AB-BA design will be used. Institutions will be randomised to start either with the intervention group (A) or with the control group (B) (TAU). After having conducted A or B, each institution will run the other condition: Institutions starting with TAU receive START NOW, and institutions that finished START NOW and the follow-up assessment conduct a new group with TAU, whereby strict rules are defined to eliminate the risk of carry-over effects.

### Study centres

The study will take place at youth welfare institutions located in proximity to four clinical trial centres. Study centres include the departments of child and adolescent psychiatry at the university hospitals in Basel, Switzerland (CS, principal investigator); Frankfurt, Germany (CMF); Aachen, Germany (BHD); and Amsterdam, The Netherlands (AP). Data management as well as biometric and statistical support are provided by the Institute of Medical Biometry and Informatics (IMBI), University of Heidelberg, Germany. The Coordination Centre for Clinical Trials (Koordinierungszentrum für Klinische Studien [KKS]) at the University of Heidelberg is in charge of study monitoring.

### Participants

Several youth welfare institutions will be asked to participate. If they agree, female adolescents in the institutions who are aged 12–20 years and their legal guardians will be asked if they want to participate. Diagnostic assessment will be standardised according to criteria of the *Diagnostic and Statistical Manual of Mental Disorders, Fourth Edition, Text Revision* (DSM-IV-TR) or the *Diagnostic and Statistical Manual of Mental Disorders, Fifth Edition* (DSM-5). Diagnostic information is obtained via a semi-structured clinical interview (K-SADS-PL). Inclusion and exclusion criteria are listed in Table 1.

**Table 1:** Inclusion and exclusion criteria

Inclusion criteria	Female adolescents aged 12–20 years
	Diagnosis of current CD (DSM code 312.8) and/or ODD (DSM code 313.81)
	Sufficient writing and reading skills (in German or Dutch)
Exclusion criteria	History or current clinical diagnosis of autism spectrum disorder according to ICD-10, DSM-IV-TR or DSM-5 (autism, Asperger syndrome, pervasive developmental disorder-not otherwise specified, atypical autism, autism spectrum disorder)
	History of or current clinical diagnosis of schizophrenia according to ICD-10, DSM-IV-TR or DSM-5
	Current clinical diagnosis of bipolar disorder or mania according to ICD-10, DSM-IV-TR or DSM-5 (History of bipolar disorder or mania is not an exclusion criterion for individuals with CD.)
	Fetal alcohol syndrome according to ICD-10, DSM-IV-TR or DSM-5
	Known monogenetic disorder, genetic syndrome (e.g., fragile X syndrome, Down syndrome, Prader-Willi syndrome)
	Any chronic or acute neurological disorder (e.g., cerebral palsy, motor problems due to motor or metabolic disorder, current treatment for epilepsy, history of severe traumatic brain injury) (Mild traumatic head injury without loss of consciousness is not an exclusion criterion.)
	Any valid indication of IQ <70 (e.g., special education, previous test results)
	Severe medical condition interfering with the intervention, including suicidal ideation
	Concurrent group-based psychotherapeutic treatment such as START NOW, DBT-A or similar interventions

*Abbreviations: CD* Conduct disorder, *DBT-A* Dialectical behaviour therapy for adolescents, *DSM-IV-TR Diagnostic and Statistical Manual of Mental Disorders, Fourth Edition, Text Revision*, *DSM-5 Diagnostic and Statistical Manual of Mental Disorders, Fifth Edition*, *ICD-10* International Classification of Diseases, Tenth Revision, *IQ* Intelligence quotient, *ODD* Oppositional defiant disorder

### Criteria for withdrawal

Participants may withdraw from the study (1) at their own request or the request of their parents/legal representatives; (2) if, according to the investigator’s opinion, participation in the trial would be harmful for the participant’s well-being; or (3) if the participant’s behaviour significantly disturbs and impedes group sessions due to, for example, aggressive behaviour directed toward other group members or herself.

### Concomitant treatment

Additional treatments administered to the participants on entry into the trial or at any time during the study are documented as concomitant treatments on the respective pages of the case report form (CRF). Concomitant treatments are allowed in the intervention group as well as the control group as long as they do not represent a group-based psychotherapeutic approach. Examples of concomitant treatments include individual psychotherapy or external support activities that serve to strengthen social-emotional, cognitive or physical competencies (e.g., drama club, chess club, soccer). Medication and changes in dosage will also be documented throughout the study.

### Measurement time points

**Fig. 1:**
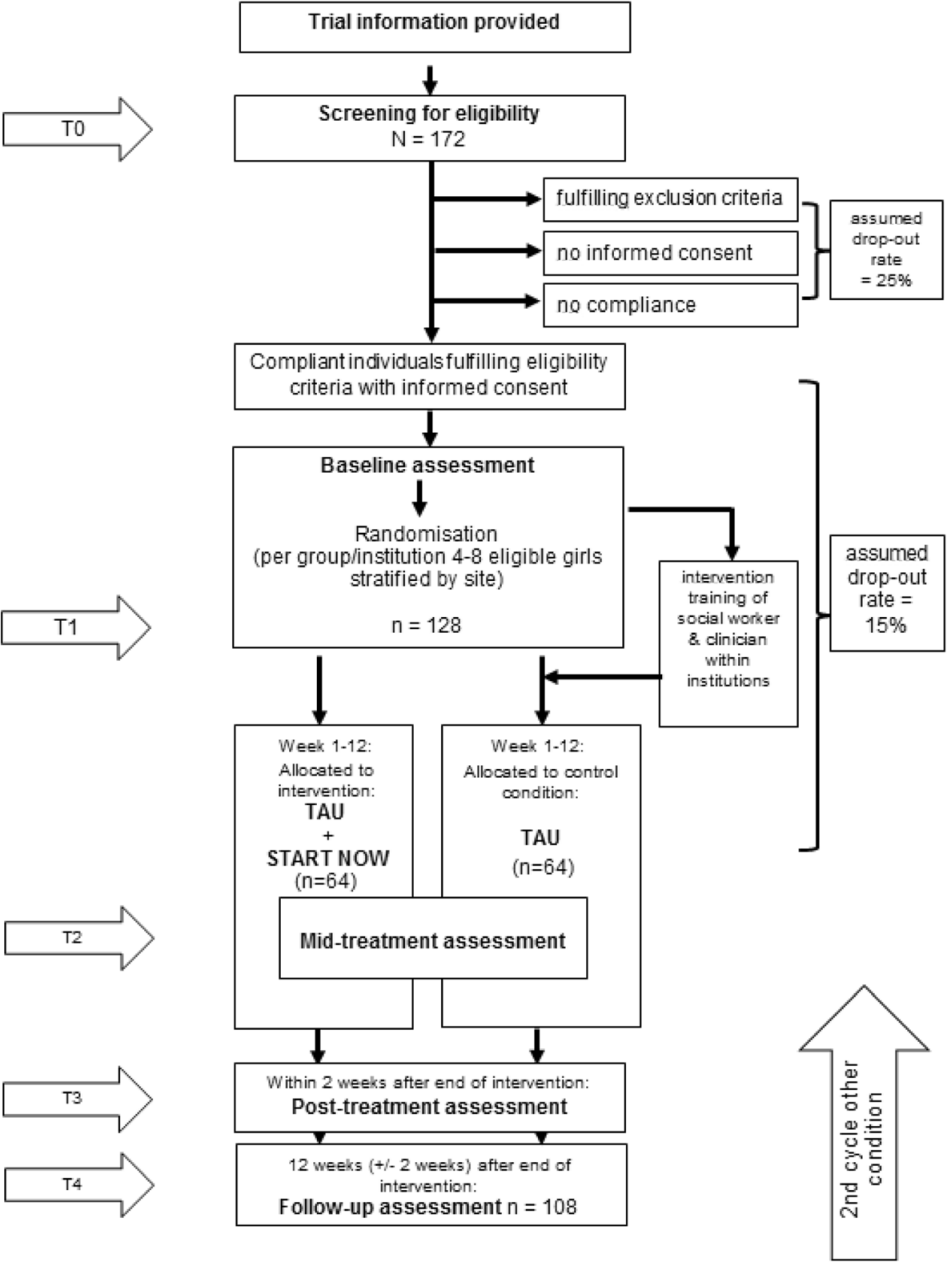
Trial flowchart. *TAU* Treatment as usual

Assessments will be conducted at baseline (T1), 6 weeks after the start of the intervention (mid-treatment [T2]), 3 months after the start of the intervention (end of treatment [T3]) and 3 months after the end of the intervention (T4). The flow of participants from recruitment through the end of the study is shown in Fig. 1. To promote participant retention, regular contact with participants through telephone calls, on-site visits and information letters is provided. Furthermore, participants who discontinue the intervention but are still interested in taking part in the data assessments are nevertheless assessed. Because the number of sessions attended is a control variable in the analysis, continuing data collection with participants who are drop-outs is not a problem.

### Primary outcome measure

The primary outcome variable is defined as change in number of fulfilled CD/ODD diagnostic criteria as assessed by a standardised, semi-structured clinical interview between baseline and post-treatment and between baseline and follow-up. The K-SADS-PL will be used [72, 73]. Interviewers will be trained by an experienced clinician, and regular consensus meetings will be held. The K-SADS-PL is a widely used and validated semi-structured diagnostic interview designed to assess current and past episodes of psychopathology in children and adolescents according to DSM-IV-TR/DSM-5 criteria. Interviews are conducted with the adolescent as well as with a social worker from the participant’s respective youth welfare institution who is not involved in the intervention. Symptom measures, diagnoses and severity ratings derived from the interview are based on the obtained information combined with clinical judgement. Thus, summary ratings are achieved by including all sources of information.

### Secondary outcome variables

Further objectives of the present trial are to assess pre-post changes in different aggression phenotypes and CD/ODD-related outcome measures in girls participating in the START NOW training compared with those receiving TAU. In adolescents, the Reactive-Proactive Aggression Questionnaire [74], used to distinguish between reactive and proactive/instrumental forms of aggression, and the Relational Aggression Questionnaire [75], which assesses relational aggression strategies such as excluding members of the peer group or spreading gossip, will be used. The Child Behaviour Checklist [76] is used to assess various externalising (attention problems, aggression, delinquency) and internalising (e.g., anxiety/depression) symptoms in adolescents via self- and parent-report. The Affective Reactivity Index (as self-rating form and as caretaker rating) [77] is used to capture general irritability. To assess psychopathic traits, the Youth Psychopathic Traits Inventory [78] will be used. Next to this self-rating questionnaire, the Inventory of Callous-Unemotional Traits, Parent Report [79], will be obtained from parents to capture change in parent-reported callous-unemotional traits. Social workers will rate aggression symptoms in daily-life situations with the modified Overt Aggression Scale originally developed by Yudolfsky et al. [80]. In adolescents also, a time-sampling short assessment procedure (ecological momentary assessment) will be used with measures collected twice daily (after lunch and after supper on 4 consecutive days; pre-, mid- and post-assessment) to capture current emotions and regulation capacities while minimising retrospection effects and interference with the daily routine. To assess change in deliberate emotional regulation strategies, the Difficulties in Emotion Regulation Scale [81] will be used.

Each female adolescent will define individual goals, and their attainment will be investigated on the basis of Goal Attainment Scaling [82]. In addition, general life satisfaction of the adolescents [83] will be assessed with the Brief Multidimensional Students’ Life Satisfaction Scale. Caretakers’ self-efficacy, burn-out symptoms and job satisfaction will be assessed with the Burnout Screening Scale, a composite instrument comprising different questionnaires [84–88]. In the intervention group, the clients’ and trainers’ satisfaction with the programme will be assessed (Client Satisfaction Questionnaire/Training Satisfaction Questionnaire) [19].

### Moderators of outcome measures

**Fig. 2:**
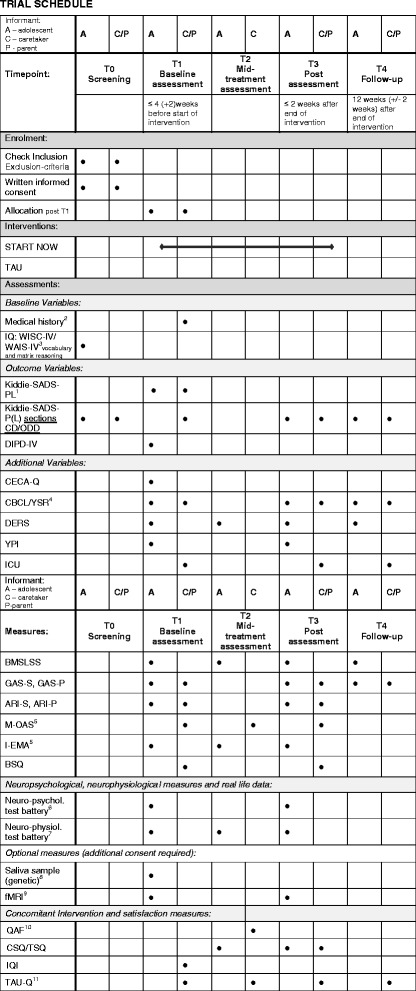
Standard Protocol Items: Recommendations for Interventional Trials (SPIRIT) checklist for the trial. *ANS* Autonomic nervous system, *ARI* Affective Reactivity Index, *BMSLSS* Brief Multidimensional Students’ Life Satisfaction Scale, *BSQ* Burnout Screening Scale, *CBCL* Child Behaviour Checklist, *CD* Conduct disorder, *CECA-Q* Childhood Experience of Care and Abuse Questionnaire, *CSQ* Client Satisfaction Questionnaire, *DERS* Difficulties in Emotion Regulation Scale, *DIPD-IV* Diagnostic Interview for DSM-IV Personality Disorders, *EMA* Ecological momentary assessment, *fMRI* Functional magnetic resonance imaging, *GAS* Goal Attainment Scaling, *HR* Heart rate, *HRV* Heart rate variability, *ICU* Inventory of Callous-Unemotional Traits, *IQ* Intelligence quotient, *IQI* Institutional Quality Index, *K-SADS-PL* Kiddie Schedule for Affective Disorders and Schizophrenia for School-Age Children–Present and Lifetime, *MOAS* Modified Overt Aggression Scale, *ODD* Oppositional defiant disorder, *QAF* Quality Assurance Form, *RSA* Respiratory sinus arrhythmia, *SCL* Skin conductance level, *SCR* Skin conductance response, *T1, T2, T3, T4* Time points 1, 2, 3 and 4, *TAU* Treatment as usual, *TAU-Q* Treatment as Usual Questionnaire, *TSQ* Training Satisfaction Questionnaire, *WAIS* Wechsler Adult Intelligence Scale, *WISC-IV* Wechsler Intelligence Scale for Children, Fourth Edition, *YPI* Youth Psychopathic Traits Inventory, *YSR* Youth Self-Report

Several potential moderators of the outcome variables will be assessed. The Massachusetts Youth Screening Instrument version 2 [89] is a self-report questionnaire that screens for a multitude of behaviour problems and potential emotional distress. The Pubertal Development Scale [90] is used to assess pubertal stage using words, rather than pictures, in those participants aged 12 years and above. The Social and Health Assessment scales [91]—specifically the subscales that relate to peers and neighbourhood environment, but also those that assess alcohol and drug use, bullying and future outlook—are used in the present study. To assess intelligence quotient (IQ), two sub-tests of the Wechsler Intelligence Scale for Children, Fourth Edition [92], or the Wechsler Adult Intelligence Scale, Fourth Edition, are performed with the adolescents: vocabulary as a measure of verbal comprehension and matrix reasoning to examine perceptual reasoning capacity. The Diagnostic Interview for DSM-IV Personality Disorders (Axis II [93]) is a semi-structured diagnostic interview done to assess the ten DSM-IV personality disorders. In the present study, a sub-section is used to assess borderline personality disorder symptoms. The quality of the youth welfare institution is systematically assessed through use of the Institutional Quality Index (IQI). The Treatment as Usual Questionnaire (TAU-Q) is distributed to assess the youth welfare institution’s standard care. Both the IQI and TAU-Q were developed specifically for the purpose of the present study. The Childhood Experience of Care and Abuse Questionnaire [94] is a self-report questionnaire that was developed to mirror an existing validated interview measure: the childhood experience of care and abuse. The questionnaire assesses lack of parental care (neglect and antipathy), parental physical abuse, and sexual abuse from any adult before age 17 years. Information regarding pre-natal and peri-natal risk factors for antisocial behaviour, such as maternal smoking in pregnancy, post-natal depression and negative living conditions in early life, as well as the socio-economic status, is additionally assessed during a standardised interview with primary caregivers. For a detailed overview on measurement time points/trial schedule, please refer to the Standard Protocol Items: Recommendations for Interventional Trials (SPIRIT) checklist shown in Fig. 2. For a detailed overview on information given within this protocol please consult (Additional file 2).

### Neurobiological measures of emotion processing

#### Neuropsychological correlates

The emotional go/no go task [95] will be used to examine regulation under multiple emotional conditions, including positive, negative and neutral facial expressions. With the Passive Avoidance Learning Test, we intend to assess stimulus-reinforcement learning (adapted from [96]).

#### Autonomous correlates

Autonomous correlates will be measured as part of a neuropsychological test battery. Basal autonomic nervous system (ANS) parameters will be measured during a 5-minute resting protocol [97]. ANS reactivity will be measured during a countdown task [98] and while watching emotion-evoking film clips [99]. Especially HRV seems to be a promising indicator of treatment success [67, 100].

#### Neural correlates

Neural correlates will be studied using functional and structural magnetic resonance imaging at baseline. Changes in these neural correlates will be investigated (in a sub-sample post-intervention in Basel and Aachen only) as mechanisms underlying the brain’s adaptation to treatments likely related to both structural plasticity and functional response alterations [101].

#### Genetic functional variants

Genetic functional variants in saliva (serotonergic, noradrenergic, dopaminergic, neuropeptide) will be investigated to test the hypothesis that specific functional genetic variants are predictive of low intervention success.

### Sample size calculation and statistical analysis

The sample size calculation is based on the primary efficacy endpoint ‘pre-post treatment change in number of fulfilled CD/ODD diagnostic criteria’ after the 12-week group-based START NOW training programme for female adolescents (as add-on therapy to TAU) compared with TAU only within youth welfare institutions. On the basis of results reported by Nelson-Gray et al. [54], pre-post changes in the treatment group of μ = 3 and in the control group of μ = 1, as well as a conservative common standard deviation of 3.5 for both groups, are expected. Because the Nelson-Gray et al. study intervention comprised only 1 DBT training module and only 16 sessions instead of 14 group sessions + 12 individual sessions as in our study, compared with the change of 2.24 in the Nelson-Gray study, we expect at least μ = 3. In addition, it has to be assumed that TAU in youth welfare settings will also result in reduction of ODD/CD symptoms. Owing to the group therapy and the respective appropriate randomisation procedure, we will have a correlated data structure. To account for that, a design effect of 1.08 to adjust for intra-group correlation of 0.02 is assumed. To achieve a power of 80% (α = 5%, two-sided; two-sample *t* test), a total of 108 patients is required for the analysis. Therefore, 172 patients will be assessed for eligibility to obtain 128 patients to be randomised (assuming that 25% of screened patients are not eligible). A drop-out rate of 15% is expected (including major protocol violations and loss to follow-up). The problem of missing values is partly resolved in the confirmatory analysis by application of an imputation strategy. Nevertheless, another 15% of patients will be randomised to compensate for the loss of information caused by drop-outs and loss to follow-up. The sample size calculation was done using nQuery Advisor 7.0 software (Statistical Solutions, Cork, Ireland).

### Statistical methods

The full analysis of the primary outcome measure will be conducted on the basis of the intention-to-treat (full analysis set) population, including all randomised patients. To account for the data structure, a linear mixed model with the primary endpoint (change in ODD/CD score between baseline and end of intervention) as the response variable, including baseline CD/ODD symptoms, group, age, site and IQ as fixed factors and the respective cluster as a random factor, will be applied. The random intercept, as well as the residuals, will be assumed to be normally distributed. The correlation structure can be referred to as compound symmetry [102], which is a common assumption in cluster-randomised trials [103]. The results will be presented as the mean between-group difference with the corresponding two-sided 95% confidence interval. The associated Hedges’ effect size *g* [104] will be calculated, in addition to the practice-related intra-cluster correlation coefficient. To fit the primary model, the restricted maximum likelihood approach will be used.

The overall type I error rate is set at α = 5% (two-sided) and will be controlled by applying the multiple test procedure for hierarchically ordered hypotheses. The first null hypothesis states that the change in ODD/CD score between baseline and end of intervention is equal for both the intervention and control groups (H_0I_: μ_1I_ = μ_2I_). This hypothesis will be tested at a two-sided level of significance of 5% against the alternative hypothesis (H_1I_: μ_1I_ ≠ μ_2I_). Only if the first hypothesis can be rejected will the second null hypothesis, which states that the change between baseline and follow-up (12 weeks after end of intervention) will be equal in both groups (H_0II_: μ_1II_ = μ_2II_), be tested in the same way. This second hypothesis can then be tested also at a two-sided level of significance of 5% against the alternative hypothesis (H_0II_: μ_1II_ ≠ μ_2II_). Application of this multiple test procedure for hierarchically ordered hypotheses assures control of the overall type I error rate of 5%. Both null hypotheses will be tested by applying a random effects model that includes baseline CD/ODD symptoms, group, age, institution and IQ as fixed factors and cluster as a random factor. This statistical analysis plan was specified before the opening of the database.

### Methods against bias

Eligibility assessment, obtaining informed consent and enrolling the participants in the trials are performed at each trial site by one of the local investigators. Institutions will be randomised to start with either the intervention group or the TAU control group. The institutions will be randomised in fully eligible groups (group size of four to eight participants), stratified by site using a centralised web-based tool (http://www.randomizer.at [14 Jan 2013], Medical University of Graz, IMBI, Statistics and Documentation, c/o Prof. Andrea Berghold). The randomisation tool is supervised by the IMBI at the University of Heidelberg, Germany.

Screening and baseline assessment are performed before the randomisation procedure, so that data obtained at intake are blinded with regard to group membership. Furthermore, all interviews will be conducted with someone who is not involved in the training.

To ensure that START NOW is implemented in all residential institutions in a comparable way, all staff trainers are trained before the beginning of the study and have to pass a practical and written test. The 2-day-long pre-training will be conducted after randomisation and before the start of the intervention. To increase treatment adherence, a facilitator manual with a detailed session-by-session outline will be used next to the workbook for adolescents. The application of the modularised sessions is strictly monitored. In addition, trainers are supervised by START NOW facilitators every other week during the intervention period. To assess adherence and competence, two training sessions of the programme (one in unit 1, one in unit 2) will be either directly attended by one START NOW supervisor or videotaped. The quality check (Quality Assurance Form [QAF] [19]) will be focused on adherence to the content of the programme and basic trainer competencies (use of motivation interviewing, rolling with resistance, validation).

With regard to the trial design, institutions that were randomised to start with the intervention condition first and subsequently provide a second cycle of TAU have to fulfil two conditions to eliminate potential carry-over effects:

1. Only social workers are involved who have not been previously trained and an exchange on START NOW content between girls is unlikely because of rare opportunity.
2. In case girls who participated in START NOW remain in the institution in the same living group after the whole cycle has ended, the time interval between the end of an intervention group and a second cycle of TAU is longer than 6 months.

Data quality is continuously monitored by the data management team (IMBI), University of Heidelberg, and the KKS). Data will be entered electronically at each local study centre site. Range checks for data values are done to promote data quality. Clinical monitors inspect subject-related data during on-site visits to ensure that all data are correct, complete and gathered according to data protection laws.

The original study forms will be kept at the trial site. Participants’ files are stored in locked cabinets, and access to the study data will be restricted. Data will be collected, processed and stored according to the data protection laws.

### Safety and ethical issues

Research activities within the FemNAT-CD START NOW project will be carried out in compliance with the fundamental ethical principles stated in the Declaration of Helsinki. The KKS, an institution that is independent from other trial staff and experienced in monitoring psychotherapy trials, will monitor the study, adapted to good clinical practice guidelines as stated by the International Conference on Harmonisation of Technical Requirements for Registration of Pharmaceuticals for Human Use, and KKS has approved the standard operating procedures. The trial (protocol version 4.1) has received approval from the local ethics committees at the University of Basel (reference EKNZ 2014/075), the University of Frankfurt (445/13 – version 5), the University of Aachen (EK 037/15) and the University of Amsterdam (2014.581-NL52038.029.14).

Any modification to the protocol will require a formal amendment and approval by each local research ethics committee. The protocol must be agreed upon by the trial steering committee (principal investigators at the Department of Child and Adolescent Psychiatry, University of Basel, and local investigators at the University of Frankfurt, University of Aachen and University of Amsterdam) and the data management team (KKS and IMBI) at the University of Heidelberg.

Participants will be enrolled only after they have received comprehensive information from the responsible investigator. Participants and their parents/legal representatives will also receive information sheets and will have the opportunity to discuss the trial with the investigator. Written informed consent for the study will be obtained by the investigator from all participants and their parents/legal representatives who are willing to participate in the trial. An example consent form is provided (see Additional file 1). All participants will be informed that they can stop participating in the study at any time without having to give justification. Participants are provided with the right to have their information removed from the database at any time. Because TAU is allowed for both the intervention the control groups, and because the control group will subsequently undergo the intervention condition, participants in either group do not experience any disadvantage with regard to group allocation in relation to clinical intervention. Control subjects who leave the institution before the start of the intervention condition are given the opportunity to participate in an outpatient START NOW group. Results will be published regardless of the magnitude or direction of the effect and will follow Consolidated Standards of Reporting Trials (CONSORT) and its extension 5 to cluster-randomised trials.

An independent data and safety monitoring board (DSMB) including three independent clinical experts and one biometrician is established to monitor the progress of the trial and ensure adherence to protocol. Safety parameters include that all serious adverse events (SAEs) or adverse events reported by the subject or detected by the local investigator that occur during the trial and all noticeable problems must be documented in the CRF. In case of an SAE, the ethics committee will be informed within 7 days after the SAE has been reported. In addition, the DSMB will also be regularly informed. In case of a significant preponderance of SAEs that might causally be associated with the trial, the DSMB will terminate the trial. Patients who are enrolled in the study are covered by indemnity for negligent harm by study insurance policies.

To guarantee confidentiality and anonymity, all participants are allocated consecutive participant identification (ID) numbers. The local principal investigator of each study site will maintain a subject ID list (participant ID codes with the corresponding participants’ names) to enable records to be identified. Authorised persons may inspect the subject-related data collected during the trial, ensuring compliance with the data protection law (inspectors, monitors, auditors). Only encoded data will be stored (centre code and consecutive ID number). Saliva samples will be stored for 30 years. Data identifying a person will be available only at the local sites. All data collected in the online database will be managed in a safe and secure environment. The system used for data management is validated and is compliant with U.S. Food and Drug Administration regulations (21 CFR part 11). All data transmission is encrypted with Secure Sockets Layer (SSL) technology. All changes to data are logged with computerised timestamps in an audit trail which includes the name of the author/editor, the date/time of the change, a reason for the change and the new information. The IMBI Heidelberg database server with the stored data is located in a secure data centre of the university and is protected by a firewall. The system provides an infrastructure to support user roles and rights. Only authorised users are able to enter or edit data, and access is restricted to data of the subjects in the respective centre.

## Discussion

This study protocol presents a randomised controlled trial testing the effectiveness of an adapted version of a cognitive behavioural skills training programme, START NOW, in institutionalised females with a diagnosis of ODD and/or CD. To date, to our knowledge, the study represents the only randomised controlled trial of the efficacy of the START NOW intervention specifically designed for female adolescents with disruptive behaviour problems within youth welfare institutions. There is an internationally recognised lack of randomised controlled studies investigating the efficacy of integrative therapeutic approaches [48, 49]. Consequently, the present study gives profound consideration to the repeatedly formulated necessity to develop integrative intervention approaches deriving from sound theoretical rationales, addressing core deficits of patients with ODD and patients with CD, and applying gender-specific strategies and materials. On the basis of convincing evidence that emotion processing is severely affected in ODD and CD, the presented approach, focused on enhancing emotional regulation and interpersonal capacities, is well-suited for this target group.

There is a specific need to implement and evaluate intervention approaches in settings where most of the affected adolescents live, most notably in residential care settings [105, 106], because residential treatment by itself seems not sufficiently effective in reducing externalising symptoms [107]. Effective treatment of severely aggressive, rule-breaking and unit-destructive behaviour is crucial to the safe operation of the residential environment [53]. This is of major relevance for staff workers dealing with adolescents who experience severe behavioural and emotional dysregulation. Thus, the implementation of evidence-based intervention approaches within youth welfare institutions is needed to strengthen the chance of continuous care and to avoid repeated relationship break-offs in severely affected adolescents living in these settings [107]. As such, the results of the present study shall be of high relevance for residential care settings and shall evoke further debates over, if not directly enhance, the standard of care provided within the youth welfare sector.

Essential methodological issues will be considered to manage threats to internal validity on the outcome of the present randomised controlled trial [108]. START NOW is manualised, and all facilitators receive 2 days of rigorous, standardised training. Beyond an in-depth preparation, START NOW’s implementation will be checked via regular fidelity monitoring assessed by the respective supervisor through use of a standard QAF. The study is large enough to determine the presence of clinically important effects, and mediators and moderators of response will be investigated. Our primary outcome measure is based on a clinical interview conducted by independent observers with both the adolescent and a (non-involved) social worker. In doing so, we gather comprehensive information that does not rely on a sole source and combines interview information, file information and clinical judgement to reach a final conclusion. Furthermore, the fact that we continuously collect neurobiological and neuropsychological data presents a major advantage because we will subsequently be able to combine and compare these objective indicators with the clinical data collected. Procedurally, the randomised AB-BA design is a further benefit because it will enable us to assess within-institution effects. This point is of particular importance because youth welfare institutions vary extremely with regard to size, type of population served, organisational and structural aspects, financial resources, and professional training opportunities. The automated online randomisation procedure is clearly defined and reflects the study design. The statistical analysis takes the different study centres and institutions into account. Furthermore, a strong advantage of the present study is the 3-month follow-up period to investigate long-term effects of the intervention.

### Limitations

A limitation of this study lies in the fact that a blind assessment can be realised only during baseline assessment. At post-assessment and follow-up, participants and other informants included in the assessments (social workers and adolescents) are aware of the condition they are in, and thus their evaluation may be biased. However, to overcome potential bias with regard to the primary outcome measure, the interview assessing CD/ODD symptoms will be conducted with social workers who are not involved in the training. In addition, to assess main secondary measures of emotion processing, objective neurobiological indicators are used which are not influenced by expectation effects.

### Trial status

In January 2015, the first group was randomised. Recruitment is expected to be finished by January 2017.

## Additional files

Additional file 1: Example informed consent form used at the Basel site. (PDF 194 kb)
Additional file 2: SPIRIT 2013 Checklist. (DOC 121 kb)

## Abbreviations

ANS: Autonomic nervous system
ARI: Affective Reactivity Index
BMSLSS: Brief Multidimensional Students’ Life Satisfaction Scale
BSQ: Burnout Screening Scale
CBCL: Child Behaviour Checklist
CBT: Cognitive behavioural therapy
CD: Conduct disorder
CECA-Q: Childhood Experience of Care and Abuse Questionnaire
CONSORT: Consolidated Standards of Reporting Trials
CRF: Case report form
CSQ: Client Satisfaction Questionnaire
DBT: Dialectical behaviour therapy
DBT-A: Dialectical behaviour therapy for adolescents
DERS: Difficulties in Emotion Regulation Scale
DIPD-IV: Diagnostic Interview for DSM-IV Personality Disorders
DSMB: Data and safety monitoring board
DSM-IV-TR: *Diagnostic and Statistical Manual of Mental Disorders, Fourth Edition, Text Revision*
DSM-5: *Diagnostic and Statistical Manual of Mental Disorders, Fifth Edition*
EMA: Ecological momentary assessment
FemNAT-CD: Neurobiology and Treatment of Adolescent Female Conduct Disorder
fMRI: Functional magnetic resonance imaging
GAS: Goal Attainment Scaling
HR: Heart rate
HRV: Heart rate variability
ICD-10: International Classification of Diseases, Tenth Revision
ICU: Inventory of Callous-Unemotional Traits
ID: Identification
IMBI: Institute of Medical Biometry and Informatics
IQ: Intelligence quotient
IQI: Institutional Quality Index
KKS: Coordination Centre for Clinical Trials (Koordinierungszentrum für Klinische Studien)
K-SADS-PL: Kiddie Schedule for Affective Disorders and Schizophrenia for School-Age Children–Present and Lifetime
MI: Motivational interviewing
MOAS: Modified Overt Aggression Scale
ODD: Oppositional defiant disorder
QAF: Quality Assurance Form
RSA: Respiratory sinus arrhythmia
SAE: Serious adverse event
SCL: Skin conductance level
SCR: Skin conductance response
SPIRIT: Standard Protocol Items: Recommendations for Interventional Trials
SSL: Secure Sockets Layer
T1: T2, T3, T4, Time points 1, 2, 3 and 4
TARGET: Trauma Affect Regulation: Guide for Education and Therapy
TAU: Treatment as usual
TAU-Q: Treatment as Usual Questionnaire
TBI: Traumatic brain injury
TSQ: Training Satisfaction Questionnaire
WAIS: Wechsler Adult Intelligence Scale
WISC-IV: Wechsler Intelligence Scale for Children, Fourth Edition
YPI: Youth Psychopathic Traits Inventory
YSR: Youth Self-Report
