# Erratum to: START NOW - a comprehensive skills training programme for female adolescents with oppositional defiant and conduct disorders: study protocol for a clusterrandomised controlled trial

**DOI:** 10.1186/s13063-017-1817-7

**Published:** 2017-03-02

**Authors:** Linda Kersten, Martin Prätzlich, Sandra Mannstadt, Katharina Ackermann, Gregor Kohls, Helena Oldenhof, Daniel Saure, Katrin Krieger, Beate Herpertz-Dahlmann, Arne Popma, Christine M. Freitag, Robert L. Trestman, Christina Stadler

**Affiliations:** 1 Department of Child and Adolescent Psychiatry/Department of Psychology, University Psychiatry Clinics Basel/University of Basel, Basel, Switzerland; 2 Department of Child and Adolescent Psychiatry, Psychosomatics and Psychotherapy, University Hospital Frankfurt, Goethe-Universität, Frankfurt am Main, Germany; 3 Department of Child and Adolescent Psychiatry, Psychosomatics and Psychotherapy, RWTH Aachen University, Aachen, Germany; 4 Department of Child and Adolescent Psychiatry, de Bascule, University of Amsterdam/VUmc, Amsterdam, The Netherlands; 5 Institute of Medical Biometry and Informatics, Department Medical Biometry, University of Heidelberg, Heidelberg, Germany; 6 Coordination Centre for Clinical Trials, University Hospital Heidelberg, Heidelberg, Germany; 7 Correctional Managed Health Care, UConn Health, Farmington, CT USA

## Erratum

The original publication [1] contains a typographical typo in the Author’s contributions section: "CS wrote the grant application." The correct sentence is: “CF coordinates the FemNAT-CD FP7 research project.”

## References

[1] START NOW - a comprehensive skills training programme for female adolescents with oppositional defiant and conduct disorders: study protocol for a clusterrandomised controlled trial. BMC Trials. 2016;17:568. DOI: 10.1186/s13063-016-1705-6 10.1186/s13063-016-1705-6 PMC5131438 27903282

