# Supplementary material for: START NOW - a comprehensive skills training programme for female adolescents with oppositional defiant and conduct disorders: study protocol for a cluster-randomised controlled trial
Source: Trials. 2016 Dec 1;17:568. doi: 10.1186/s13063-016-1705-6 (PMC5131438; doi:10.1186/s13063-016-1705-6)
Supplement: Additional file 1: — Example informed consent form used at the Basel site. (PDF 194 kb) [file 13063_2016_1705_MOESM1_ESM.pdf]

Kinder- und Jugendpsychiatrische Klinik

Prof. Dr. phil. Dr. med. Dipl. Psych. Christina Stadler
Professorin für Entwicklungspsychopathologie
Schanzenstrasse 13
CH-4056 Basel
Tel: +41 61 685 21 24
Fax: +41 61 685 21 68
christina.stadler@upkbs.ch

UPK Universitäre Psychiatrische Kliniken Basel

**Nummer der Studie:**	
**Titel der Studie:**	„Gruppentrainingsprogramm für Mädchen mit Störungen des Sozialverhaltens: Die zentrale Rolle der Emotionsregulation" (Kurztitel FemNAT-CD-Intervention)"
**Sponsor**:	EU
**Ort der Studie:**	Basel, Deutschschweiz
**Prüfer:**	Prof. Dr. phil. Dr. med. Dipl. Psych. Christina Stadler
**Teilnehmerin**<br>Name und Vorname:<br>Geburtsdatum:<br>Adresse:<br>Telefon:	

Sehr geehrte Damen und Herren

Liebe Jugendliche

Dies ist die Einverständniserklärung zur Teilnahme an einer klinischen Studie.
Bitte lesen Sie dieses Formular sorgfältig durch und fragen Sie, wenn Sie etwas nicht verstehen oder erläutert bekommen möchten.

- Ich wurde vom unterzeichnenden Arzt/Psychologen/Wissenschaftler mündlich und schriftlich über die Ziele der Studie, den Ablauf, über die zu erwartenden Wirkungen und mögliche Vor-/Nachteile und Risiken der oben genannten Studie informiert.
- Ich habe die zur oben genannten Studie abgegebene schriftliche Information vom 10.02.2016, Version 3.01, gelesen und verstanden. Meine Fragen im Zusammenhang mit der Teilnahme an dieser Studie sind mir zufriedenstellend beantwortet worden. Ich kann die schriftliche Information behalten und erhalte auf Wunsch eine Kopie meiner schriftlichen Einverständniserklärung.
- Ich hatte genügend Zeit, um meine Entscheidung zu treffen.
- Ich bin darüber informiert, dass Schäden gedeckt sind, falls solche im Rahmen der Studie auftreten.
- Ich nehme an dieser Studie freiwillig teil. Ich kann jederzeit ohne Angabe von Gründen meine Zustimmung zur Teilnahme widerrufen, ohne dass mir oder meinem Kind deswegen Nachteile bei der weiteren medizinischen Betreuung entstehen.
- Ich bin einverstanden, dass die zuständigen Fachleute der Studie und der Ethikkommission zu Prüf- und Kontrollzwecken in die Originaldaten Einsicht nehmen dürfen, jedoch unter strikter Einhaltung der Vertraulichkeit. Ich bin damit einverstanden, dass Testungen, welche bereits innerhalb einer Klinik oder während eines stationären Aufenthaltes in Verbindung mit der UPK Basel durchgeführt wurden, mit dem Forschungsteam geteilt werden können, sodass keine (unnötigen) Doppeltestungen stattfinden müssen.
- Ich bin mir bewusst, dass während der Studie die in der Information für Versuchspersonen genannten Anforderungen und Einschränkungen einzuhalten sind. Im Interesse der Gesundheit meines Kindes kann der Prüfarzt mein Kind jederzeit von der Studie ausschliessen. Zudem informiere ich den Prüfarzt über die gleichzeitige Behandlung meines Kindes bei einem anderen Arzt sowie über die Einnahme von Medikamenten (vom Arzt verordnete oder selbständig gekaufte).

- Ich wurde über die Möglichkeit eines Zufallsbefundes bezüglich der Gesundheit meines Kindes aufgeklärt und erkläre mich mit meiner Unterschrift in diesem Dokument bereit, dass wir darüber in Kenntnis gesetzt werden und eine Abklärung bei einer Fachstelle oder durch einen qualifizierten Arzt empfohlen wird.
- Ich weiss, dass meine persönlichen Daten und die meines Kindes nur in verschlüsselter Form an aussenstehende Institutionen zu Forschungszwecken weitergegeben werden. Ich bin einverstanden, dass die Behörden und die Kantonale Ethikkommission zu Prüf- und Kontrollzwecken in meine Originaldaten Einsicht nehmen dürfen, jedoch unter strikter Einhaltung der Vertraulichkeit.

☐ Ich möchte nicht an der Bildgebungsstudie teilnehmen.

☐ Ich möchte nicht an dem schulähnlichen Leistungstest teilnehmen.

☐ Ich möchte nicht an der genetischen Untersuchung teilnehmen.

☐ Ich möchte nicht an der hormonellen Untersuchung teilnehmen.

☐ Ich möchte nicht, dass Videoaufnahmen von 2 Sitzungen des Gruppentrainings gemacht werden.

☐ Ich möchte nicht, dass Angaben, die in den letzten 12 Monaten bereits in der KJPK erhoben wurden, verwendet werden.

Ort, Datum	Unterschrift der Mutter
Ort, Datum	Unterschrift des Vaters
Ort, Datum	Unterschrift gesetzliche Vertretung
Ort, Datum	Unterschrift von Jugendlichen ab 14 Jahren

Ich bin damit einverstanden, dass ich **zu einem späteren Zeitpunkt** von den Studienleitern erneut kontaktiert werde (z.B. für Folgefragen oder erneute Studienteilnahme):

Ort, Datum	Unterschrift der Mutter
Ort, Datum	Unterschrift des Vaters
Ort, Datum	Unterschrift von Jugendlichen ab 14 Jahren

Bestätigung des Prüfers:
Hiermit bestätige ich, dass ich den Eltern und/oder Erziehungsberechtigten sowie Ihrem Kind Wesen, Bedeutung und Tragweite der Studie erläutert habe. Ich versichere, alle im Zusammenhang mit dieser Studie stehenden Verpflichtungen zu erfüllen. Sollte ich zu irgendeinem Zeitpunkt während der Durchführung der Studie von Aspekten erfahren, welche die Bereitschaft des Patienten zur Teilnahme an der Studie beeinflussen könnten, werde ich umgehend darüber informieren.

Ort, Datum	Unterschrift der Prüfers
